# Supplementary material for: Vaccines safety and maternal knowledge for enhanced maternal immunization acceptability in rural Uganda: A qualitative study approach
Source: PLoS One. 2020 Dec 10;15(12):e0243834. doi: 10.1371/journal.pone.0243834 (PMC7728220; doi:10.1371/journal.pone.0243834)
Supplement: S1 File — (PDF) [file pone.0243834.s001.pdf]

FOCUS GROUP DISCUSSION (FGD) GUIDE

PREGNANT WOMEN

Selection criteria: Pregnant women, who have used or not used vaccines

Copies of informed consent and confidentiality forms should be provided to each participant and read aloud for the benefit of those who cannot read. Participants should be provided an opportunity to ask any questions.

The following is a guide. Try to ask all the questions below in the order given, but it is more important to maintain the flow of discussion. Suggested probes have been included.

You should try to encourage participation of all group members in the conversation.

Start by explaining the ground rules as follows:

Before we start, I would like to remind you that there are no right or wrong answers in this discussion. We are interested in knowing what each of you think, so please feel free to be frank and to share your point of view, regardless of whether you agree or disagree with what you hear. It is very important that we hear all your opinions.

You probably prefer that your comments not be repeated to people outside of this group. Please treat others in the group as you want to be treated by not telling anyone about what you hear in this discussion today. Let's start by going around the circle and having each person introduce herself. (Members of the research team should also introduce themselves and introduce the topic of vaccination and immunization and describe each of their roles.)

1. What do you think about vaccination and immunization?
2. Let’s talk about some **experiences** you have had with vaccines and vaccination. Who would like to share?
    - What have you heard about other women’s experiences with vaccination?
    - Some women in the community do not want to receive vaccines. Why do you think that is?
    - Have you heard of any woman with negative effects from vaccine? What about yourself?
    - Do you know anyone who has had a vaccine-preventable disease? Can you share that person’s story?

3. What vaccines do you **know** that are routinely or occasionally received by women here in Uganda?
    - Have you heard of any new vaccines and, if so, what have you heard?
    - Why would someone in your community refuse or hesitate to receive vaccination- probe distance, timing, knowledge, etc.?
    - What are modes of administration of vaccines do you know? Which ones do you prefer? Probe Nasal, oral and injection.
4. What are some of the **cultures, norms and beliefs** that influence vaccinations in your community?
    - Probe for community leaders, historical influences and media environment
    - Policies and politics that influence vaccination
    - Probe for religions and parental norms (pregnancy before marriage) and gender roles in the community
    - Belief that Vaccine Preventable Disease are needed to build immunity or vaccines are associated with reducing fertility rates, or destroy important Natural immunity.
    - Belief that traditional medicines are important than vaccination to prevent Vaccine Preventable Disease.
5. Where do you get **information** related to vaccines?What sources related to vaccines do you think are most trustworthy, and why? Probe for health workers, media and social networks?
    - Are there some opinion leaders who have influenced you to vaccinate?What about those who have discouraged you to vaccinate?
    - What would be the best or easiest wayfor you to learn more about vaccination?

6. Do think that a **vaccination schedule** is flexible enough to allow you vaccinate with convenience?
    - Probe for multiple vaccines and age of vaccines (Hep B)
    - Have you ever gone to the health facility and you did not get vaccination? Why was this?
    - Do you think that the health facilities have the vaccines that you could demand for to prevent vaccine preventable diseases?

7. If a **new vaccine** were introduced into the country, would you be willing to take up the vaccine, what would you want to know about the vaccine?
    - How would you recommend such a vaccine to be delivered? Probe house to house or campaign, by government or at Health centers & timing.
    - What do you think people need to know in order to trust vaccination?
8. What do you think about the **distance** from your home to the place of immunization?
    - How much do you spend to get to the hospital? Do some people think it is far?
9. Tell me about your experience with **health workers** on vaccination? Do you think they trust the vaccines?
    - Probe waiting time, hospital environment, and interpersonal relations with Health workers.
10. Let's **summarize** some of the key points from our discussion.
    - This has been a very good discussion! Is there anything else anyone would like to say about vaccines and vaccination?

Thank you for taking the time to talk to us!
