# Supplementary material for: Vaccines safety and maternal knowledge for enhanced maternal immunization acceptability in rural Uganda: A qualitative study approach
Source: PLoS One. 2020 Dec 10;15(12):e0243834. doi: 10.1371/journal.pone.0243834 (PMC7728220; doi:10.1371/journal.pone.0243834)
Supplement: S2 File — (PDF) [file pone.0243834.s002.pdf]

KEY INFORMANT INTERVIEW GUIDE:

HEALTH WORKERS

Selection criteria: Health Workers, who are working in immunization Unit

Copies of informed consent and confidentiality forms should be provided to each participant. Participants should be provided an opportunity to ask any questions.

The following is a guide. Try to ask all the questions below in the order given, but it is more important to maintain the flow of discussion. Suggested probes have been included.

You should try to encourage participation of the informant to keep the conversation going.

1. What do you think about vaccination and immunization?
2. Let's talk about some **experiences** you have had with vaccination of pregnant women and children.
    - Some pregnant women in the community do not want to receive vaccines. Why do you think that is?
    - Have you heard of any pregnant woman with negative effects from vaccine?
    - Do you know anyone who has had a vaccine-preventable disease and it is directly attributed non vaccination? Can you share that person's story?
3. What vaccines do you **know** that are routinely or occasionally received by pregnant women here at this health facility?
    - Have you heard of any new vaccines and, if so, what have you heard?
    - Why would someone in your community refuse or hesitate to receive vaccination-probe distance, timing, knowledge, etc.?
    - What are modes of administration of vaccines do you know? Which ones do pregnant women prefer? Probe Nasal, oral and injection.
4. What are some of the **cultures, norms and beliefs** that influence vaccinations in this community?
    - Probe for community leaders, historical influences and media environment
    - Policies and politics that influence vaccination
    - Probe for religions and parental norms (pregnancy before marriage) and gender roles in the community
    - Belief that Vaccine Preventable Disease are needed to build immunity or vaccines are associated with reducing fertility rates, or destroy important Natural immunity.

- Belief that traditional medicines are important than vaccination to prevent Vaccine Preventable Disease.

5. Where do pregnant women get **information** related to vaccines? What sources related to vaccines do you think are most trustworthy, and why? Probe for health workers, media and social networks?
    - Are there some opinion leaders who have influence women to vaccinate? What about those who have discouraged people from vaccinating?
    - What would be the best or easiest way for you to learn more about vaccination for women?
6. Do think that a **vaccination schedule** is flexible enough to allow women vaccinate with convenience?
    - Probe for multiple vaccines and age of vaccines (Hep B)
    - Are there times when women go to the health facility and they do not get vaccination? Why was this?
    - Do you think that the health facilities have the vaccines that yourself could demand for to prevent vaccine preventable diseases?
7. If a **new vaccine** were introduced into the country, would you be willing to take up the vaccine, what would you want to know about the vaccine?
    - How would you recommend such a vaccine to be delivered? Probe house to house or campaign, by government or at Health centers & timing.
    - What do you think people need to know in order to trust vaccination?
8. What do you think about the **distance** of the place of immunization to the people you serve?
    - How much do you think is the average expenditure for most of the women to get to the hospital for vaccination? Do some people think it is far?
    - This has been a very good discussion! Is there anything else anyone would like to say about vaccines and vaccination?

Thank you for taking the time to talk to us!
