# Supplementary material for: Vaccines safety and maternal knowledge for enhanced maternal immunization acceptability in rural Uganda: A qualitative study approach
Source: PLoS One. 2020 Dec 10;15(12):e0243834. doi: 10.1371/journal.pone.0243834 (PMC7728220; doi:10.1371/journal.pone.0243834)
Supplement: S3 File — (PDF) [file pone.0243834.s003.pdf]

Thematic classification of additional quotes from participants.

Quotes	Themes and sub-themes
	**Knowledge bout Maternal vaccines**
**Quote 1a**	**Beneficial to mother & children**<br>"*As pregnant women…., we are vaccinated Tetanus because a mother may deliver along the road and this vaccination for Tetanus saves the mother. Even when you deliver at home, the umbilical cord should be handled carefully and does not get Tetanus because of the vaccine injected during pregnancy" (FGD7).*
**Quote 1b**	**Prevention of malaria and general body weakness**<br>*"What I think about vaccination is that it prevents diseases like tetanus and others like malaria, body weakness…" (FGD 4) "…it helps babies not to be attacked by malaria" (FGD 6)*
	**Attitudes and beliefs towards maternal vaccines**
**Quote 2a**	**Personal responsibility**<br>*"The life of someone is very important. You can stop doing anything and attend to health workers and fulfil what he/she told you. So, I say that you have to go" (FGD 1).*
**Quote 2b**	**Traditional remedies and Religious beliefs**<br>*"…. they use local herbs (Namuvu and emumbwa) and they take it every day" (FGD 5).*
	"...... others fail due to religious faith that do not allow them to go for vaccination like the born again (Ngiri nkalu) (FGD 4).
**Quote 2c**	**Fear of injection and HIV testing**<br>*"…others fear tetanus injection and to be tested for HIV/AIDS." (FGD 9).*
	**Experiences of pregnant women with maternal vaccines**
**Quote 3a**	**Scheduling**<br>*"The vaccination schedule is really convenient because it helps them to get vaccinated at the time they have come for ANC…."*(KII 3).
	"When you go early there is no problem but when you go late, they will tell you to come on the next day since they will be tired already." (FGD 3)
**Quote 3b**	**Waiting time**<br>"*Women who come for ANC are always very many and you have to work for long hours to complete all of them and it is tiring to…."*(KII 4).
**Quote 3c**	**Harsh and abusive health workers**<br>"*Some health workers abuse patients telling them that they are dirty."* (FGD 1).
	**Willingness to receive new vaccine**
**Quote 4a**	**Sensitized of vaccine**<br>"As *long as we are sensitized about the new disease which is affecting pregnant women such that we can abide with the new vaccine (FGD 5).*
	"I am ready because it will help us pregnant women and our babies in the womb" (FGD 6).

**Quote 4b**	**AEFI concerns**<br>*"If the vaccine has no side effect to the baby and the mother, I will take it……." (FGD 5).*
	"…..she told that her husband said that if it (vaccine) can cripple an old child, then how about the unborn one…"(KII 1).
**Quote 4c**	**Guinea pigs for vaccines**<br>*"For us as people, we are prepared but the problem is that they tell us that [the President of Uganda] wants to test the drugs on us the Ugandans from this side so it's after finding out that the drug has no problem, that is when it's taken to his home area for administration" (FGD 2).*
	**Partner involvement in decision making for maternal vaccination**
**Quote 5a**	**Partners as reminders**<br>*"My husband reminds me when I forget…" (FGD 8).*
**Quote 5b**	**Partners as for ANC**<br>*"I always go with my husband for ANC most of the time but in…" (FGD 8)*<br>*"We encourage them to come with their husbands while attending ANC since they help….."*(KII 1).
**Quote 5c**	**Dress code and hygiene**<br>*"They are dirty. One says I will not go because I will be a laughingstock since my husband didn't buy me maternity dress and I have rugs (FGD 4).*
