# Supplementary material for: Vaccines safety and maternal knowledge for enhanced maternal immunization acceptability in rural Uganda: A qualitative study approach
Source: PLoS One. 2020 Dec 10;15(12):e0243834. doi: 10.1371/journal.pone.0243834 (PMC7728220; doi:10.1371/journal.pone.0243834)
Supplement: S5 File — (PDF) [file pone.0243834.s005.pdf]

Codebook for Analysis

Themes	Sub-themes	CODES	Meaning
Knowledge about maternal vaccination	Correct knowledge	Know_Cor_prev_Mother	Correct knowledge that vaccines prevent mothers from diseases like Tetanus
		Know_Cor_Prev_Baby	Correct knowledge that vaccines prevent children from diseases e.g. measles, tetanus and correct information about immunization improving immunity
		Know_CC_Vac	Knowledge of cervical cancer vaccine
		Know_Hep.B_Vac	Knowledge of Hepatitis B vaccine
	Wrong Knowledge	Know_Wrg_dfd_Baby	Wrong/ Incorrect Knowledge/information about vaccination that it prevents delivery of deformed babies
		Know_Wrg_Malaria	Incorrect knowledge that vaccination prevents malaria
		Know_Wrg_Weakness	Wrong info/knowledge that vaccination protects from general body weakness
	No knowledge	Noknow_Vac	Lack of knowledge about vaccination e.g. which vaccine is given and when
Attitudes and beliefs towards Maternal Vaccination	Health system/ Environment factors	NoVac_Poor_attd_H/W	Harsh/abusive /unbothered health workers & long stay at H/F
		NoVac_trspt	Long distances and lack of transport
	Individual factors	NoVac_Reluc	Because of reluctance e.g some keep postponing. some say since i deliver from home, no ANC need doing & other chores like gardening
		NoVac_HIVtest	Fear of testing for HIV
		NoVac_men	Being stopped by men for due to over crying at night after vaccination, fear of death of the child
		NoVac_fear_Expry	Fear of being injected expired vaccines and wrong drugs
		NoVac_fear_Side Effect	Fear of side effects: becoming lame, unable to lift arm after injection
		NoVac_fear_inject	Fear of injections/ tablets
		NoVac_notSick	No need for vaccination as they don't feel sick
		NoVac_trdmed/hlrs_reli	Opt for traditional healers/herbal medicine & some religious faith e.g. born again "Ngiri nkalu" prohibit and TBAs
		NoVac_Maternity_Dress	Husbands' failure to buy maternity dresses and escorting them

Themes	Sub-themes	CODES	Meaning
	Worries about AEFI for Maternal vaccination	NoVac_ sid_eff_fever	Side effect: child injected, got fever
		NoVac_ sid_eff_wound	Side effect: injected and area developed pus/became wound
		NoVac_ sid_eff_disabled	Side effect: injected and child became disabled
		NoVac_sid_eff_Parlysd+pain	Side effect of painful paralyzed are of injection
		NoVac_side effects_death	Side effect of death after vaccination
Experiences with Maternal Vaccination	Schedule	Exp_Schd_Busy	The schedule is busy
		Exp_Schd_ltd	The time schedule is limited and not convenient
		Exp_Schd_convinient	The schedule and the time is enough and convenient
	Maternal Vaccination logistics and Amenities	Exp_Vac_refrige	Not vaccinated due to faulty refrigerator
		Exp_Vac_Stockout	Not vaccinated due to drug/vaccine stock outs
		Exp_poorSan	Experience of poor sanitation at facility; dirty toilets
		Exp_Vac_prescribed	Prescribe for you in the book to buy if no drugs
		ExP_lngWaiting time	Experience of long waiting hours
	Experiences of Vaccination with Health Workers	Exp_Rude_ H/W	Experience with harsh/ rude/abusive health workers
		Exp_good trt	Experience of good treatment by the health workers (especially when you go early)
		Exp_no_H/W	Experience of finding no health workers around: when they have left especially after mid-day
		Exp_H/W_bribe	Experience some health workers favoring bribe
		Exp_NoVac_late	Experience of not being vaccinated by health workers because of reaching late
Readiness to receive Maternal vaccines	Readiness & willingness & Issues of concern	NewVac_Ready_no problem	Ready to take up the vaccine (majority kept on insisting if it has no problem)
		NewVac_NoReady_wait	First wait for other people to be vaccinated
		NewVac_diff Btn_O/N	Need to know the difference btn new and old vaccine
		NewVac_Sen_Side eff.	Need sensitization about the vaccine's side effects (preferably no side effect)
		NewVac_Import.	Want to know what the vaccine cures/ prevents against/vaccine importance
	Preference for delivery of Vaccine	Prefer_ inject	Prefer an injection
		Prefer_ Mouth	Prefer mouth drops
		Prefer_ tabs	Prefer tablets

Themes	Sub-themes	CODES	Meaning
		Prefer_ H/F	At facility level
		Prefer_D2D	Door to door
		Prefer_Outreach	Community outreaches
		Prefer_Village	At village level e.g. the chairperson's home, VHT
		Dist_Long	The distance is long
		Dist_short	The distance is short
	Information sources	Info_VHTS	VHTS
		Info_Media	Media e.g TV, RADIO
		Info_Opn ldrs	Local council leaders e.g. The village women leader (nabakyaala)
		Info_Frds	Friends
		Info_Soc_gather	Social gatherings like burials
		Info_H/W	Health workers
		Info_Dist	HDSS from District offices
		Info_Van	Moving vans/community mobilisers
		Info_Wors_Places	Religious leader and places of worship
Male/ partner involvement in maternal vaccination	Involvement of partners	Part_enc_Vacc	Partners encourage their pregnant women to go for vaccination
		Part_Sup_Vac	Partners offer direct support to partners including transport and going with them for maternal vaccination and ANC
		Part_notSup_Vac	Partners are not engaged in direct support for pregnant women to vaccinate
			Partners worry about the side effects of maternal vaccines while citing experiences
