# Supplementary material for: Vaccines safety and maternal knowledge for enhanced maternal immunization acceptability in rural Uganda: A qualitative study approach
Source: PLoS One. 2020 Dec 10;15(12):e0243834. doi: 10.1371/journal.pone.0243834 (PMC7728220; doi:10.1371/journal.pone.0243834)
Supplement: Attachment — Submitted filename: Review of manuscript on maternal immunization.docx [file pone.0243834.s006.docx]

**Review of manuscript for PLOS ONE**

**Summary:** This is a well done manuscript. The title, background, findings, and discussion are very well laid out. Methods need some clarity for reproducibility. Just a few suggestions to make it a little more crips:

**Comments on**

**Title:** No comment

**Abstract:**

Lines 33 &34: You need to recast this sentence. “Women expressed willingness to take new vaccines, positive attitudes towards maternal immunization, and were familiar with its importance”. Recast the sentence to follow the order in your objectives namely knowledge, attitudes and willingness. For example: “Women were familiar with the importance of maternal vaccines, have positive attitudes and expressed willingness to take them”.

Line 35: “……... affected by adverse events”, ie changing the effects to events. Also recast the statement to be more sharp and crisp. Such as “………. affected by worries of pregnant women and that of their partners’ who influence health seeking decisions in a home concerning adverse events following the matenal immunization.”

Line 36-38: “Misconceptions about introduction of vaccines like thinking that vaccines will be tested on only them before introduction in larger population, and that vaccines treat illnesses like malaria and general body weakness.” Consider this phrase “ …… of vaccines such as the belief that vaccines treat malaria and general body weakness and that of being used as guinea-pig to test for the vaccine before its introduction to the larger population.”

**Background:**

Line 77: It is World Health Organization and not “world ….”

Line 80: “………. are administered to pregnant women.”

Line 82: Do you meen that poor neonatal health outcomes are due to lack of studies on knowledge ………. Make it clearer.

Line 87: Be consistent. “ Immunisation” is British English. You have been using American English. In addition, there is a little flaw in the content and mechanical accuracy in that sentence. You could consider “This can partly be attributed to limited knowledge among pregnant women, their poor attitude about immunization, their failure to attend all ANC visits, lack of training of the Village Health Teams (VHTs) on the importance of TT vaccination for pregnant mothers and limited health education to pregnant mothers.”

Line 92: “ ………. middle-income countries in respect of Pneumococcal Conjugate Vaccine ……….. “

Line 95: GAVI means Global Alliance for Vaccines and Immunizations.

**Methods:** This section need some work.

Line 144: You had included health workers in the research population included. You failed to include them now.

Line 153: Which of the population had FGD and which one had KII. You need to be coherent and orderly.

Line 157: Readers may find it difficult to understand what you mean. I think you may look at it again and recast it to bring out your points clearly.

Line 165: Order and sequence is important as mentioned in previous comment.

Line 174: Pointless mentioning names of those that played a role here.

**Results:**

Line 194: “ (2 FGDs pregnant women and 2 health workers) could read “(2 FGDs for pregnant women and 2 KIIs for 2 health workers)”, or what exactly, do you have in mind.

Line 208: Change the FDG to FGD. Where exactly is the stuty area; rural or urban or rural and semi-urban? Be consistent.

Line 239: You use either “reason” or “why”.

Line 250: I could have preferred “….one woman added” than the word chipped in. This is scientific writing.

Line 264: AEFI means adverse events following immunization. It refers to any untoward medical occurrence which follows immunization and which *does not necessarily have a causal relationship* with the usage of the vaccine.

Line 283: could read “ …… highlighted that some of ……”

Line 290: “Participants also expressed that some pregnant women did not go for vaccination due to their prohibitive religious practice like some Pentecostal churches”, could read “Participants …….. due to their religious belief that prohibites vaccination”.

Line 296: This statement “Some mentioned the fear for injections and HIV testing (which is compulsory for all women attending ANC) would be the reason why some women do not seek maternal vaccination” could read “Fear of injections and HIV testing could be the reason some women fail to seek maternal vaccination.”

Line 369: “When health workers were asked what women are worried about, only one mentioned AE worries from the pregnant women and their partners” could read “When health workers were asked what women are worried about, only one mentioned that the pregnant women and their partners were concerned about AEFI.”

Line 375: The statement could be rephrased thus “Some participants believe that they were used to test for the safety of new vaccines and thus they would not take the vaccines until its safety is confirmed by others who had received it.

Line 389: Could be rephrased as “Pregnant women …………. vaccination even without accompanying them and their children to immunization centers.”

Line 400: You may want to rephrase thus “Health workers cited that they encouraged pregnant women to come with their partners for ANC visits.”

Line 417: “…………. not effective since women are not comfortable with moving with husbands and more so their husbands don’t dress well.”

**Discussion:**

Line 434: be consistent with the study area.

Line 452: change adverse effect to adverse event

Line 475: Rephrase as “…………... and religious practices found in some Pentecostal churches, that prohibite maternal vaccination.

Line 482: Rephrase the statement for clarity

Line 486: Rephrase the statement. It could read “Some believe that the new vaccines …………...”

Line 502: delete treatment from the sentence.

Line 504: You can rephrase as “The attitudes and behaviors of health care providers towards pregnant women is an …….”

Line 517: Consider rephrasing thus, “……… get vaccinated for their good and that of their children”.

Line 518: Consider this rephrasing “The partner’s worries about AEFI was an important concern for women ………….”

Line 537: See the comment on line 34

Line 538: Define ADR. Do you mean Adverse Drug Reaction? Since we are talking of immunization why not use AEFI instead.
