# Supplementary material for: Vaccines safety and maternal knowledge for enhanced maternal immunization acceptability in rural Uganda: A qualitative study approach
Source: PLoS One. 2020 Dec 10;15(12):e0243834. doi: 10.1371/journal.pone.0243834 (PMC7728220; doi:10.1371/journal.pone.0243834)
Supplement: Attachment — Submitted filename: Response to Reviewer 2.docx [file pone.0243834.s009.docx]

**Point by Point Response to Review of manuscript**

Reviewer #2: Good job. You just need to re-read the article painstakingly for few grammatical errors, most of which I have pointed out. See the attachment

**Response: Thank you very much for the guidance and pointing out the grammatical errors. We have reviewed the manuscript, corrected the errors, clarified where necessary and improved the flow of the manuscript.**
